# A rapid HPLC-FLD method for Ochratoxin A detection in pig muscle, kidney, liver by using enzymatic digestion with MISPE extraction

**DOI:** 10.1016/j.mex.2020.100873

**Published:** 2020-03-19

**Authors:** Giacomo Luci

**Affiliations:** Department of Clinical and Experimental Medicine, University of Pisa, Via Roma 55, 56126 Pisa, Italy

**Keywords:** OTA, Solid phase extraction, Liquid chromatography

## Abstract

Ochratoxin A (OTA) is a mycotoxin produced as a secondary metabolite by various Aspergillus and Penicillium species with nephrotoxic, carcinogenic, immunotoxic and teratogenic effects. OTA has been found in several food commodities, including cereals, beer, wine and spices. OTA can also be present in animal products (especially in pig derived products) as a result of carryover from contaminated feed. Permitted level of 1 µg/kg OTA in pig meat or pig-derived products was set in Italy, as in other countries. Key parameters which affected MISPE, should be described such as extraction efficiency and were optimized, analyzed by an isocratic HPLC-FLD method. Under the optimized conditions, for all analyzed matrices mean recovery was > 89%. Method can be applied as alternative routine procedure to detect OTA presence in pig products.

Points:

*Aim of the study was to develop and validate a quantitative HPLC-FLD method based on MISPE with complex solid matrices (edible tissues) followed by chromatographic analysis.

*The new method was developed and validated in pig complex matrix and is very sensitive LOD and LOQ respectively 0.001 and 0.003 µg/kg.

*This method is relatively simple to use and with good performances. Was possible to reuse MISPE column with a “regeneration” solution, until to 7 times.

## Graphical abstract

**Figure:**
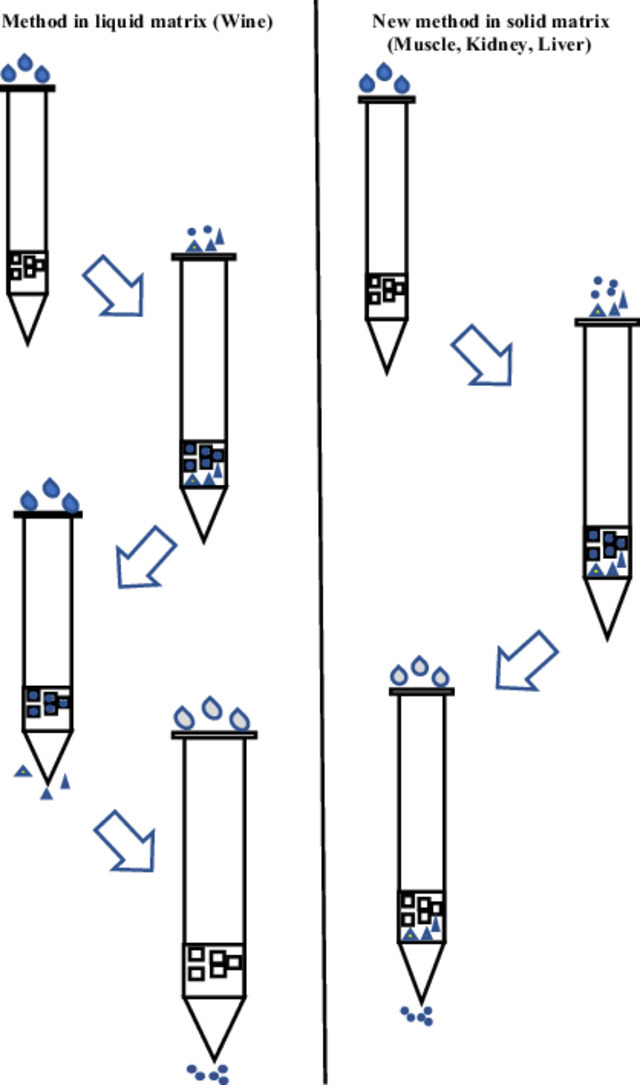

**Table:** Specifications table

Subject Area	Veterinary Science and Veterinary Medicine
More specific subject area	*Veterinary Toxicology and food safety*
Method name	*Molecularly Imprinted Solid Phase Extraction method with HPLC-FLD detection*
Name and reference of original method	*“Selective Solid Phase Extraction of Ochratoxin A from Wine Products Using Molecularly Imprinted Polymers”*
Resource availability	*The link reference is:* http://www.affinisep.com/media/application_note_affinimip_spe_ochratoxin_a_red_and_white_wine__280411__098591900_1514_01092011.pdf

## Method details

Mycotoxins are secondary metabolites naturally produced by molds. Ochratoxin A (OTA) is a mycotoxin produced as a secondary metabolite by various *Aspergillus* and *Penicillium* species with nephrotoxic, carcinogenic, immunotoxic and teratogenic potential [1]. OTA is widely distributed in various food commodities, including cereals, coffee, beer, wine and spices. As cereals are widely used in animal feed, animals can be exposed to OTA through the consumption of contaminated feed, which can lead to the accumulation of this mycotoxin in their tissues [2]. Ranging OTA levels of 0.1–1 µg/kg have been detected in foodstuffs of animal origin (pork and chicken meat, dry-cured ham) [2], [3], [4]. Although no European Union (EU) maximum levels for OTA in food of animal origin have been laid down, some countries have set maximum levels of OTA in pigs meat or derived products, such as Denmark (pig kidney 10 µg/kg, pig blood 25 µg/ml), Estonia (pig liver 10 µg/kg), Romania (pig kidney, liver and meat 5 µg/kg) and Slovakia (meat 5 µg/kg, milk 5 µg/kg) [5]. Others such as Italy have developed guidelines for recommended maximum OTA levels (pig meat and derived products 1 µg/kg) (Italian Ministry of Health, 1999), [6].

OTA is suspected to cause the Balkan Endemic Nephropathy, which is a fatal kidney disease observed in rural areas of southeast Europe [7]. Human epidemiology studies in several countries including Italy have found significantly higher serum or plasma levels of OTA in patients with certain kidney disorders compared to healthy people, although the association may not be a causal one [8]. Similar studies are reported in dogs and pigs [9], [10], [11]. Due to its ubiquitous presence in foodstuffs and their potential risk for human health its effective detection is deemed important.

OTA identification in biological samples generally involved liquid-liquid extraction, clean-up by an immunoaffinity column (IAC), and identification by HPLC with fluorescence detection (HPLC-FLD) [1]. Various extraction and clean-up procedures for the determination of OTA by HPLC-FLD in pigs tissues and derived products has been reported: extraction with chloroform/ethyl acetate, IAC clean-up, solid phase extraction (SPE) with C18 [12], [13], [14], [15], [16]. IAC purification is simple, rapid, and provides high OTA recoveries and quite repeatable results. Unfortunately, the IACs are relatively costly and have a short shelf life. SPE is an extraction method used for pre-concentration and clean-up of biological and environmental samples. It is simple and rapid and consumes little amount of organic solvent. Despite these characteristics, the classical SPE sorbents (C8, C18, and so on) retain molecules by nonselective hydrophobic reaction, which leads to the co-extraction of interfering substances and further purification techniques are still required to remove co-eluted compounds [17]. Therefore, new sorbents such as molecularly imprinting polymers (MIPs) are progressively more developed to meet the need of high selectivity. The application of MIPs to SPE has also enhanced the selectivity for extraction of analytes from complex matrices. MIPs have been used to detect OTA in wheat, wine, beer, grape juice and coffee coupled to HPLC-FLD [18], [19], [20], [21], [22].

The aim of the present study was to develop a new enzymatic digestion method coupled with molecularly imprinted solid phase purification (MISPE) for quantitative determination of OTA in pig tissues (muscle, liver, kidney), for to be applied as alternative routine procedure to detect OTA presence in pig meat products, in order to make lab work planning easier [23], or applied to dosing samples for future applications, as already done in other fields: compared with reference method [24] steroidogenesis effects of OTA [25,26], genetics effects with new technique applications [27] or correlate OTA levels with GFR (Glomerular Filtration Rate) valutation of pigs, with different sampling [28,29].

## Materials and methods

### Reagents

Ochratoxin A (from *Aspergillus ochraceus*) (MW = 403.8) was purchased from Sigma-Aldrich (Darmstadt, Germany). Stock solution was prepared in toluene/acetic acid (99:1 v/v) at concentration of 200  µg/l and stored at −20 °C. Working OTA standard solutions were prepared by diluting the stock solution with the HPLC mobile phase. HPLC-grade water, methanol, ethylacetate and acetonitrile were purchased from VWR (Milan, Italy). The pancreatin enzyme (from porcine pancreas) was purchased from Sigma (code P1750, Darmstadt, Germany), and was stored at – 20 °C until use. MISPE columns (product code: Affinimip® SPE OCHRATOXIN A – 6 ml, 125 mg sorbent), were purchased by Affinisep (Petit-Couronne, France).

### High performance liquid chromatography instrumentation

The chromatographic system consisted of a Perkin Elmer (Waltham, USA) Series 200 binary pump and a Jasco FT-1520 fluorescence detector (Jasco, Tokyo, Japan). The excitation wavelength was set at 380 nm and emission wavelength at 420 nm. Totalchrom Navigator® software was used for data processing. The reversed-phase column was a C_18_ HAISIL HL, (5 mm × 150 mm, 4.6 mm; Higgins Analytical, USA). The column was kept at room temperature. The HPLC was operated with a mobile phase system consisting of a methanol-phosphate buffer solution pH 7.5 (0.03 M Na_2_HPO_4_, 0.007 M NaH_2_PO_4_) 50/50% v/v at flow rate of 1 ml/min.

### Enzymatic digestion

Muscle, liver and kidney samples were digested according to Luci [30] with slightly modifications. Briefly, 5 g of sample were homogenated with Ultra turrax T-25 homogenizer in 5 ml of phosphate buffer solution pH 7.5 (0.2 M Na_2_HPO_4_, 0.2 M NaH_2_PO_4_ 84/16% v/v), digested with 10 ml of pancreatin 1% (p/v) in phosphate buffer solution pH 7.5 (0.2 M Na2HPO4, 0.2 M NaH2PO4 84/16% v/v) for 1 h at 37 °C, under shaking conditions. The digested samples were applied on MISPE columns after ultrasonication applying cycles (75 Hz) of 1 min for 15 min with sample at 0 °C (Ultrasonics Cheimika® model FS/000300/N – 300 W – 5:200 ml – Salerno, Italy)

### Optimization of purification procedure in MIP modified column (MISPE-approach optimization)

The protocol suggested by Affinisep [31] (http://www.affinisep.com/media/application_note_affinimip_spe_ochratoxin_a_red_and_white_wine__280411__098591900_1514_01092011.pdf) (Table 1) for MISPE procedure of OTA in wine samples was changed to optimize the purification parameters for pig tissues samples. Pig muscle, liver and kidney samples of 10 animals were obtained from local slaughterhouses. Samples were frozen at −20 °C until analysis

**Table 1:** MISPE extraction conditions as reported in [31] and in the present study.

MISPE step	Extraction protocol of OTA from wine samples [31]	Optimized developed protocol of OTA from pig tissues
Conditioning of the column	3 ml acetonitrile, 3 ml of deionized water	3 ml acetonitrile, 3 ml of deionized water
Loading	Maximum 10 ml of sample diluted 1:1 (v/v) with HCl solution (pH=1; 0,1 M)	10 ml of digested sample
Washing	6 ml 60/40 HCl solution pH=1; 0,1 M / Acetonitrile (v/v)	No washing step
Elution	3 ml Acetic acid / Methanol 2/98 (v/v)	6 ml Acetic acid / methanol 2/98 (v/v)

### Conditioning and loading

MISPE columns were conditioned with 3 ml acetonitrile, then with 3 ml of deionized water as reported in Affinisep protocol for wine samples (Table 1). Muscle, liver and kidney digested and ultrasonicated samples (initial volume 5 or 10 ml) were diluted 1:1 before being loaded on columns with HCl solution (pH = 1; 0,1 M). Muscle, liver and kidney digested and ultrasonicated samples (initial volume 5 or 10 ml) were also loaded on columns without any dilution and adjusted to pH 2–3 with H_3_PO_4_ 85%.

### Washing and eluting

On MISPE columns were tried the completely dried under vacuum and, other option, washed with 6 ml of 60/40 HCl solution pH=1, 0.1 M /acetonitrile (v/v). Finally, columns were tried to elute with 3, 6, 12 or 24 ml of acetic acid/methanol 2:98 (v/v). The extract was dried under gentle nitrogen flow and reconstituted in 500 µl of HPLC mobile phase.

### Reusability of MISPE

The reusability of MISPE was also investigated. 10 consecutive clean‐up cycles were performed on a single MISPE column for muscle, kidney and liver samples (1 µg/kg). After each cycle, the column was washed with 5 ml of methanol and 5 ml of water for the regeneration of polymers, which could avoid carry‐over effects between the individual cycles.

### Enzymatic digestion coupled to liquid partition

Spiked tissues samples were digested and purified by liquid-liquid partition method to compare the obtained relative chromatograms and the recovery values with those obtained by using optimized MISPE extraction procedure. Samples were extracted according to [30]. Briefly, ethyl acetate extracts of digested samples were centrifuged, evaporated with nitrogen, resuspended in HPLC mobile phase and analyzed by HPLC.

### Method validation

A recommended maximum OTA level of 1 µg/kg (1 ppb) in pork meat and derived products was established by the Italian Ministry of Health in 1999 [6]. The validation procedure was performed considering the value of 1 µg/kg OTA.

The analytical method was validated according to [32] by evaluating: specificity, recovery, trueness, decision limit (CCα), detection capability (CCβ) of the method selectivity, linearity, LOD and LOQ, repeatability and reproducibility.

The linearity of the method was evaluated by analyzing calibration curve samples, prepared by spiking muscle, liver and kidney samples with OTA at 0.05, 0.1, 0.5, 1, 2.5 and 5 µg/kg. Samples were extracted with the MISPE protocol and analyzed using the HPLC method. Considering the concentration step during MISPE protocol, these spiked samples corresponded to OTA standard concentrations of 0.00125, 0.0025, 0.0125, 0.025, 0.0625 and 0.125 µg/ml. The experiment was repeated 3 times. The correlation coefficient (r^2^) and the goodness-of-fit coefficient (g) were determined and were required to be ≥0.99 and ≤10.00%, respectively.

Intra-day precision and accuracy was evaluated by analyzing 3 muscle, liver and kidney samples spiked with OTA at low (0.1 µg/kg), medium (1.0 µg/kg) and high (5.0 µg/kg) concentration levels, extracted with the MISPE protocol and analyzed using the HPLC method on the same day. Considering the concentration step during MISPE protocol, these spiked samples corresponded to OTA standard concentrations of 0.0025, 0.025 and 0.125 µg/ml. Inter-day precision and accuracy was evaluated by analyzing the same samples on different days (*n* = 7). The acceptance criteria for accuracy were −20% to +10% of the theoretical concentration. For the precision, the relative standard deviation (RSD) was defined as RSDmax = 2^(1−0.5logConc)^  ×  2/3 for within-day precision and 2^(1−0.5logConc)^ for between-day precision.

The LOQ was defined as the lowest OTA concentration which the method was validated with a precision and accuracy that fell within the recommended ranges (see intra-day accuracy and precision). The LOQ was also established as the lowest point of the calibration curve. The LOQ was determined by analyzing 6 spiked blank muscle, liver and kidney samples on the same day.

The LOD was defined as the lowest OTA concentration which could be measured by the fluorimetric detector with a signal-to-noise (S/N) ratio of ≥3. The LOD values were calculated by comparing the S/N ratios in the LOQ and blank muscle, liver and kidney samples.

CCα, defined as the concentration at and above which it can be concluded with an error probability of *α* (*α* = 5%) that a sample is non-compliant or with statistical certainty of 1 – *α*, was estimated analyzing 18 (6 for each matrix) spiked samples with 1 µg/kg of OTA. The concentration at this limit plus 1.64 times the corresponding standard deviation equals the CCα (*α* = 5%). CCβ, defined as the concentration at which method is able to detect the permitted limit with a statistical certainty of 1−*β* (*β* = 5%), was determined by analyzing 18 (6 for each matrix) spiked at the CCα level The value of CCα plus 1.64 times the corresponding standard deviation equals the CCβ (*β* = 5%).

## Results and discussion

The need for fast, reliable and low-cost analytical methods to monitoring OTA in food and biological samples could be based on easy sample pre-treatment coupled to simple analytical protocol. For this reason, we developed an original sample pre-treatment protocol, based on MISPE columns, coupled to the HLPC-FLD analysis, aiming to simplify the OTA extraction procedure from pig's tissues samples.

In the present study, the critical variables affecting the SPE (nature and pH of loading solution, nature and volume of washing solution and volume of eluting solution) were optimized using pigs tissues samples spiked with OTA.

**Fig. 1:**
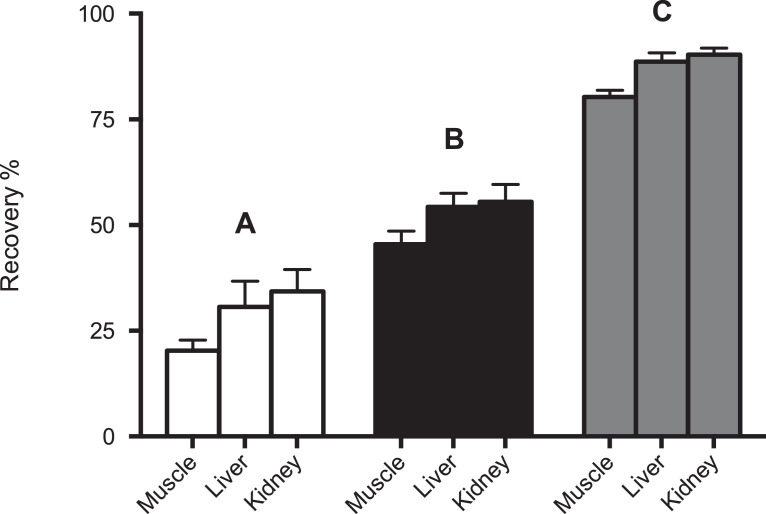
Recovery (%) of OTA after passing 10 ml of pig muscle, liver and kidney digested samples spiked at 1 ppb (*n* = 3) and (A) diluted 1:1 with HCl solution (pH = 1; 0.1 M); (B) adjusted to pH 2–3 with H_3_PO_4_ 85%; (C) ultrasonicated applying cycles (75 Hz) of 1 min for 15 min with sample at 0 °C.

In the protocol by Affinisep for wine samples dilution 1:1 with HCl solution (pH = 1; 0.1 M) of samples and the loading of a maximum of 20 ml of sample have been indicated for the optimal extraction of OTA. This mycotoxin have high binding affinity to proteins such as blood serum albumin, thus producing an easy-to-accumulate molecule in tissues and organs. In the present study, digested samples with pancreatin because, this proteolytic enzyme, can destroy tissues and release OTA in pancreatin solution. After this step, solution couldn't be loaded directly to MISPE columns due to presence of precipitate and column clogging, but after ultrasonication, resulted clear and without precipitates. The dilution of ultrasonicated digested samples with HCl resulted in recoveries < 40%, while the adjustment of ultrasonicated digested samples to pH to 2 by using H_3_PO_4_ 85% showed recoveries < 60%. When 10 ml of ultrasonicated digested samples without any dilution solvent were passed through the column the recovery was >80% for all analyzed matrices (Fig. 1).

**Fig. 2:**
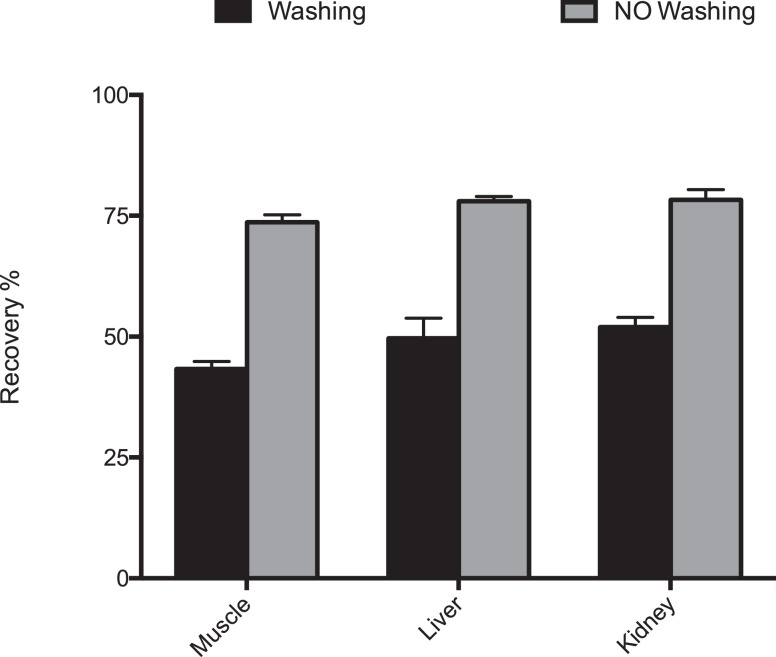
Recovery (%) of OTA washing or without washing solvents after passing 10 ml of pig muscle, liver and kidney digested samples spiked at 1 ppb (*n* = 3) and ultrasonicated applying cycles (75 Hz) of 1 min for 15 min with sample at 0 °C.

**Fig. 3:**
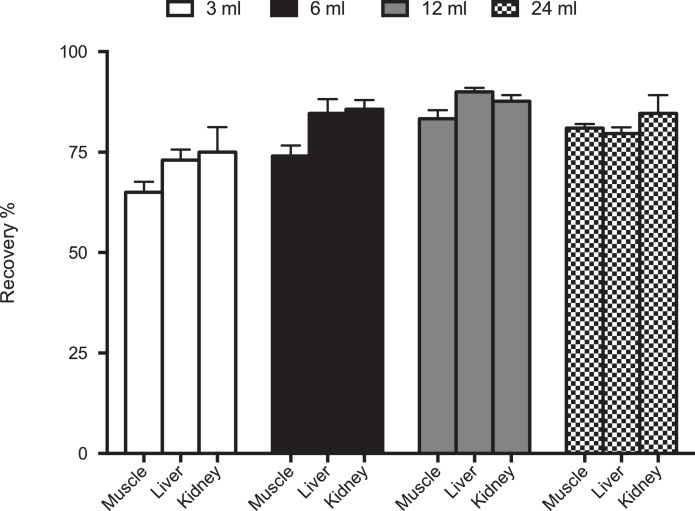
Recovery (%) of OTA using different samples volumes of elution buffer after passing 10 ml of pig muscle, liver and kidney digested samples spiked at 1 ppb (*n* = 3) and ultrasonicated applying cycles (75 Hz) of 1 min for 15 min with sample at 0 °C.

An evaluation regarding the washing step of the MISPE extraction was conducted. In MISPE protocol by Affinisep for wine samples the washing is applied to remove the unbound material from the column, and because off the high affinity of the analyzed molecules towards molecularly imprinted materials with this step a very good cleanup can be achieved with respect to conventional SPE. In the present study, it was found that washing with 6 ml of 60/40 HCl 0.1 M/Acetonitrile (v/v) reduced the recovery of OTA from all the analysed matrices whereas best recoveries were obtained without washing step (Fig. 2). This is probably due to the nature of the matrix analyzed (homogenated tissues samples) containing mainly nonpolar interferents compounds, while in wine sampls are also present polar compunds. Finally, column elution was carried out by using different volumes of acetic acid/methanol 2:98 (v/v), 12 ml of elution solution gave the best peak area and recoveries compared to the other volumes and it was chosen for the subsequent experiments (Fig. 3). The adopted MISPE conditions are reported in Table 1.

The MISPE device can be reused up to 7 times without any significant reduction in peak area (not shown). Although reusability has been claimed also for IAC, the complete regeneration of the selective binding properties was found to be challenging and was reported to require extended incubation (>10 h) in special buffers at low temperatures [33] MISPE columns can be cleaned and reactivated under relatively harsh conditions for repeated uses due to their high chemical robustness.

**Fig. 4:**
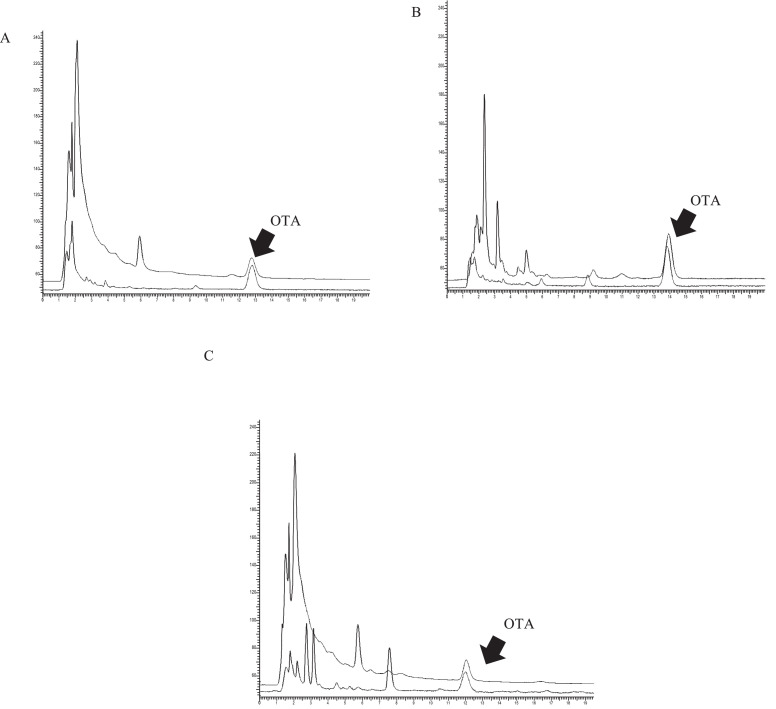
Typical HPLC‐FLD chromatograms of (A) pig muscle sample spiked with OTA at the level of 1 µg/kg after clean‐up with liquid–liquid partition (upper line) and MISPE (lower line); (B) pig kidney sample spiked with OTA at the level of 1 µg/kg after clean‐up with liquid-liquid partition (upper line) and MISPE (lower line); (C) pig liver sample spiked with OTA at the level of 1 µg/kg after clean‐up with liquid–liquid partition (upper line) and MISPE (lower line).

The analytical features of the newly developed method were compared with ethyl acetate clean-up procedure [30]. The methods were found to have similar sensitivity, linear range and recoveries. The chromatograms obtained by applying the developed HPLC method to spiked tissues samples purified with ethyl acetate and optimized MISPE procedure are shown in Fig. 4. The use of the MISPE purification reduced significantly the matrix interferences of all the matrices analyzed.

The new developed method was compared with the other reported methods in the same matrices (Table 2). The LOD and LOQ values obtained are lower compared to an earlier HPLC-FLD work that was developed for muscle, kidney and liver using liquid-liquid extraction (LLE) and dispersive liquid-liquid microextraction (DLLME) as sample preparation [12,^14^,15]. It is also interesting to note that the sensitivity of the proposed method (reflected in LOD and LOQ) is better than the more expensive LC–MS/MS methods [12,13] due to high selectivity and sensitivity of the fluorescence detection at longer excitation and emission wavelengths. Validation parameters are reported in Table 3. The range of linearity for OTA was 0.05 to 5 µg/kg for all the matrices studied. The linearity obtained by linear regression showed values of r^2^ > 0.99. The intra-day variability was <4.93%, while the inter-day was < 3.95%. The LOQ and LOD of the method were 0.003 and 0.001 µg/kg, respectively, for all the matrices studied.

**Table 2:** Comparison of the developed method with the previous study for the determination of OTA in pigs tissues.

Instrument	Extraction method	Type of sample	Repeatability (%RSD)	Recovery (%)	LOD (μg/kg)	LOQ (μg/kg)	Ref.
HPLC-FLD^a^	MISPE^d^	Muscle, kidney, liver	0.12–4.93	86.00–98.00	0.001	0.003	Current study
	LLE^e^	Muscle, kidney	8.00–15.00	74.00–86.00	0.140	0.520	[15]
	DLLME^f^	Muscle	2.60–4.40	88.00–92.00	0.210	0.700	[14]
	LLE^e^	Kidney, liver	–	71.00	0.140	0.250	[12]
UPLC/MS/MS^b^	IAC^g^	Kidney	4.60–7.50	74.00–92.00	0.030	0.100	[13]
LC-MS/MS^c^	LLE^e^	Kidney, liver	–	77.00–89.00	0.250	0.500	[12]

a High performance liquid chromatography coupled with fluorescence detector.

b Ultra high performance liquid chromatography – tandem mass spectrometry.

c Liquid chromatography–mass spectrometry.

d Molecularly imprinted polymer solid phase extraction.

e Liquid–liquid extraction.

f Dispersive liquid–liquid microextraction.

g Immunoaffinity column extraction.

**Table 3:** Validation parameters for the analysis of OTA in pigs muscle, kidney and liver samples by using molecularly imprinted polymers solid-phase extraction protocol coupled with HPLC-FLD method.

Sample	Linear range (μg/kg)	*R*^2^		LOD (μg/kg)	LOQ (μg/kg)
Muscle	0.05–5	0.998 ± 0.002		0.001	0.003
Liver	0.05–5	0.996 ± 0.003		0.001	0.003
Kidney	0.05–5	0.999 ± 0.002		0.001	0.003
Spiked sample	Recovery (% ± SD)	Intra-day (%RSD)	Inter-day (%RSD)	CCα (μg/kg)	CCβ (μg/kg)
Muscle					
0.1 µg/kg	86.11 ± 3.54	3.01	3.95	1.031	1.060
1 µg/kg	94.00 ± 3.29	3.80	3.50		
5 µg/kg	98.70 ± 0.92	1.00	0.93		
Liver					
0.1 µg/kg	94.50 ± 2.67	2.11	2.82	1.069	1.059
1 µg/kg	93.33 ± 2.50	2.75	2.68		
5 µg/kg	98.63 ± 1.10	0.40	1.11		
Kidney					
0.1 µg/kg	93.50 ± 3.39	4.93	3.63	1.100	1.098
1 µg/kg	93.68 ± 3.67	4.40	3.92		
5 µg/kg	98.67 ± 0.80	0.12	0.81		

## Conclusions

A new simple method based on molecularly imprinted polymer as selective SPE sorbents has been established for the determination of OTA in pig muscle, kidney and liver samples by HPLC coupled with fluorescence detection, and the validated method shows satisfactory linearity, precision and accuracy. MISPE showed simplicity, reusability and longer storage time and can be considered as an alternative to the commonly used IAC technique for the sample clean-up and pre-concentration. The results indicated that the established method could be applied for trace analysis of OTA in a complex sample matrix.

## Declaration of Competing Interest

The author declares that he has no conflict of interest.

## Appendix Supplementary materials

Image, application 1
